# Effects of High-Temperature Exposure on the Mechanical Properties of Kenaf Composites

**DOI:** 10.3390/polym12081643

**Published:** 2020-07-23

**Authors:** Nabilah Afiqah Mohd Radzuan, Dulina Tholibon, Abu Bakar Sulong, Norhamidi Muhamad, Che Hassan Che Haron

**Affiliations:** 1 Department of Mechanical and Manufacturing Engineering, Faculty of Engineering and Built Environment, Universiti Kebangsaan Malaysia, Bangi 43600, Selangor, Malaysia; dulina@gmail.com (D.T.); norhamidi@ukm.edu.my (N.M.); chase@ukm.edu.my (C.H.C.H.); 2 Department of Mechanical Engineering, Politeknik Merlimau, KB 1031, Pejabat Pos Merlimau, Merlimau 77300, Melaka, Malaysia

**Keywords:** fibers, mechanical properties, mechanical testing, compression molding

## Abstract

Automotive parts, including dashboards and trunk covers, are now fabricated through a compression-molding process in order to produce lightweight products and optimize fuel consumption. However, their mechanical strength is not compromised to avoid safety issues. Therefore, this study investigates kenaf-fiber-reinforced polypropylene composites using a simple combing approach to unidirectionally align kenaf fibers at 0°. The kenaf composite was found to withstand a maximal temperature of 120 °C. The tensile and flexural strengths of the aligned kenaf composites (50 and 90 MPa, respectively) were three times higher than those of the commercialized Product T (between 39 and 30.5 MPa, respectively) at a temperature range of 90 to 120 °C. These findings clearly showed that the mechanical properties of aligned kenaf fibers fabricated through the combing technique were able to withstand high operating temperatures (120 °C), and could be used as an alternative to other commercial natural-fiber products.

## 1. Introduction

Sustainability and ecofriendliness are the current main criteria for manufacturing products [1]. Composite materials, therefore, seem suitable to fulfilling the requirements for producing ecofriendly products, given that the mechanical properties of these materials are easily customized to manufacturers’ needs [2,3]. However, commonly applied composite materials, including glass- and carbon-reinforced polymer, are nonbiodegradable, with high processing costs and the need to comply with environmental legislation, limiting their application and use [1,4]. Given these limitations, natural fibers, known as biocomposites, were introduced as an alternative to existing composite materials because they offer good biodegradability, low density, and they have adequate mechanical properties [5]. International automotive manufacturers have incorporated these natural fibers into most manufactured automotive parts, allowing 80 wt % of waste to be recycled and reused [1,6]. However, none of these biocomposites is available for high-temperature products, given that natural fibers are usually unstable at temperatures above 100 °C, thus reducing the strength of the composites by around 10% every 10 min [7,8].

This study proposes that the kenaf fiber, as a biodegradable material in conjunction with a conventional manufacturing process, can withstand operating temperatures as high as 120 °C. Kenaf stems consist of bast and core fibers known as kenaf fibers. Bast and core fibers exhibit a strength of 20 to 230 MPa with an average density of 1.45 g/cm^3^, while the kenaf fiber itself can reach a maximal tensile strength of 930 MPa [9,10,11]. However, the main drawback of using kenaf fibers is their hydrophilic nature, as they cannot be mixed with the hydrophobic polymer matrix given the issues surrounding their adhesion qualities [12,13]. Hence, the researchers of this study tried to address and overcome this issue, including by using a surface treatment of 4 wt % of NaOH, which improved their mechanical properties by 78% [10,14]. Previous studies demonstrated that, by using kenaf-fiber-reinforced epoxy resin and aligning the fibers in a particular direction (0° to 90°), their mechanical properties could be improved without using any surface treatment. Moreover, by diminishing the surface-treatment procedure, a more affordable and time-effective manufacturing process could be introduced [15].

Meanwhile, in the conventional manufacturing process, industries often use either injection-or compression-molding techniques to fabricate composite mixtures [16]. However, those techniques result in randomly oriented kenaf fibers, which further reduces their mechanical properties, with an average strength of 30 MPa [17]. Currently, natural composites are often used as an interior part, in which their mechanical properties can be compromised. However, recent studies have indicated that, through certain processes, these mechanical properties can be improved. Hence, these processes can be implemented elsewhere, for example, in dashboard panels and trunk covers. Prior research exhibited that adapting the compression-molding process aids in developing an aligned kenaf composite [18]. Thus, it strengthens the mechanical properties of kenaf composites, as its performance can be altered according to manufacturers’ needs. However, the main concern in using kenaf composites in dashboard or trunk covers is their ability to withstand high temperatures (above 60 °C). Thus, in addition to fabricating the kenaf composite through a compression-molding process, a discussion on fiber orientation was also crucial, as it has consequent effects on the composite’s mechanical strength. The importance of fiber orientation was also supported by previous studies in which composite materials that exhibited desirable mechanical properties were dependent on composite structure and orientation angle [8,19]. Therefore, on this basis, orientation studies were conducted by the researchers of this study to enhance the mechanical properties of kenaf composites, especially under high-temperature operations. Some studies also indicated that the fiber orientation of composite materials frequently results in warpage, which degrades their mechanical performance as temperatures increase [20,21]. Hence, this study focused on the effect of temperatures ranging from 30 to 120 °C on the different orientations of kenaf-reinforced polypropylene (PP) composites.

## 2. Methodology

### 2.1. Materials

**Figure 1:**
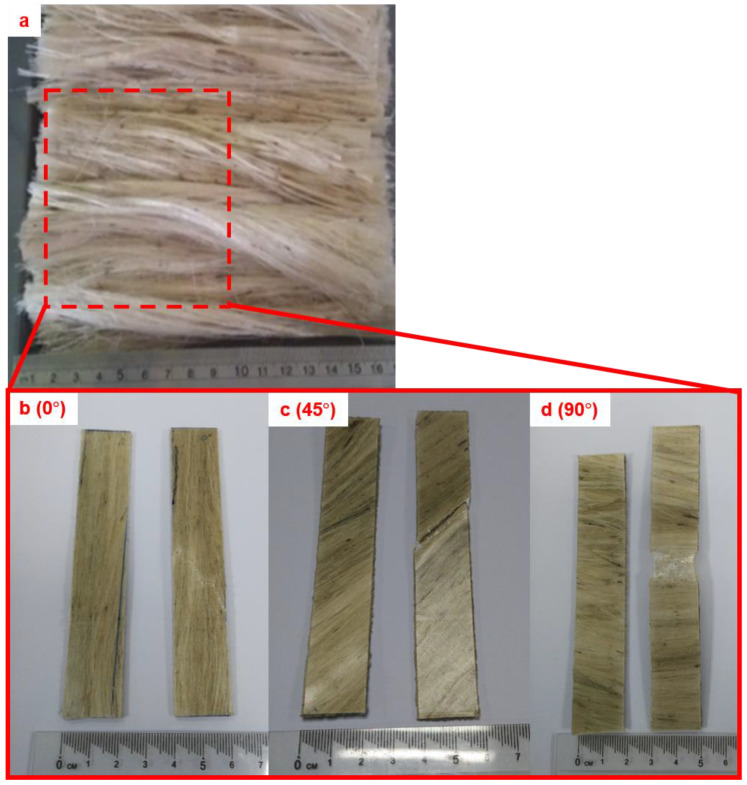
(**a**) Kenaf fibers supplied by Lembaga Kenaf dan Tembakau Negara (LKTN), as received. Kenaf composite at different orientation angles of (**b**) 0°, (**c**) 45°, and (**d**) 90°.

Kenaf fibers (bast), as shown in Figure 1a, had an average length between 350 and 400 mm, and were supplied by Lembaga Kenaf dan Tembakau Negara (LKTN, Kelantan), Malaysia. This type of kenaf was selected on the basis of its ability to exhibit excellent mechanical properties without requiring to undergo an alkaline-chemical-treatment process [21]. Details on the chemical composition of the kenaf fibers are listed in Table 1. The thermoplastic polymer used in this study was polypropylene, grade Titan-600, in powder form with an average size of 90 µm, a density of 910 kg/m^3^, and a melting temperature of 160 °C. It was supplied by Goonvean Fibres Ltd., England, UK.

### 2.2. Fabrication Process

The kenaf bast fibers initially underwent a water-retting process for 7 to 10 h to separate the outer layer (bast) from the inner layer (core), ensuring that the kenaf-fiber surface was clean of any impurities. The water-retting process enhances the surface roughness of the kenaf fiber, which aids in the adhesion of polymer resin onto its surface [22]. The kenaf bast fibers were later combed, which indirectly removed any physical impurities on the kenaf fibers’ surface, to ensure that the fibers would be long, smooth, and continuous before drying in an oven at 40 °C for 24 h. The prepared kenaf fibers were then cut into 175 mm long pieces/strips, and aligned on 0°, 45° and 90° angles before being placed in the hot-press mold (175 × 175 × 2 mm), as shown in Figure 1b–d. The kenaf fibers were then sandwiched together with the polypropylene powder; 40 wt % of kenaf and 60 wt % of polypropylene were used. After that, the prepared materials underwent a compression-molding process using a 50 ton hot-press machine at a pressing temperature between 190 and 210 °C, and with applied pressure between 5 and 10 MPa for between 2 and 6 min.

### 2.3. Characterization

The obtained samples were then cut into pieces with a width of 20 mm, a length of 115 mm, and a thickness of 2 mm for the tensile test based on ASTM D638-99. The tensile test was conducted using the Zwick 30 kN AllroundLine attached to a tube furnace, with the temperature set at 30, 60, 90, and 120 °C, respectively. Next, the flexural-strength test was conducted using the Zwick 30 kN with a speed of 2 mm/min. The dimensions of the sample were 70 × 20 × 2 mm based on ASTM D790-99 for this specific test. Both the tensile and flexural tests were performed using 10 sets of samples. Dynamic mechanical analysis (DMA) was performed using the PerkinElmer DMA 8000 machine at room temperature until a temperature of 150 °C was reached. This test required the samples’ dimensions to be 30 × 10 × 2 mm based on the ASTM D5023 standard, with the heat rate set at 2 °C/min, and with the frequency set at 1 Hz. Unlike with the DMA test, Poisson’s ratio was determined using a Kyowa CC-33A and a PCD 300 Series sensor surface on the basis of ASTM D638-99. Poisson’s ratio was then monitored using the PCD 300B software that was attached to the Zwick 30 kN AllroundLine machine. A total of five samples were used for each conducted test. Poisson’s ratio was measured on the basis of the Young’s modulus and shear modulus using Equation:

$$v=\frac{E}{2G}-1,$$

where *v* is Poisson’s ratio, *E* is Young’s modulus, and *G* is the shear modulus [23].

## 3. Results and Discussion

**Figure 2:**
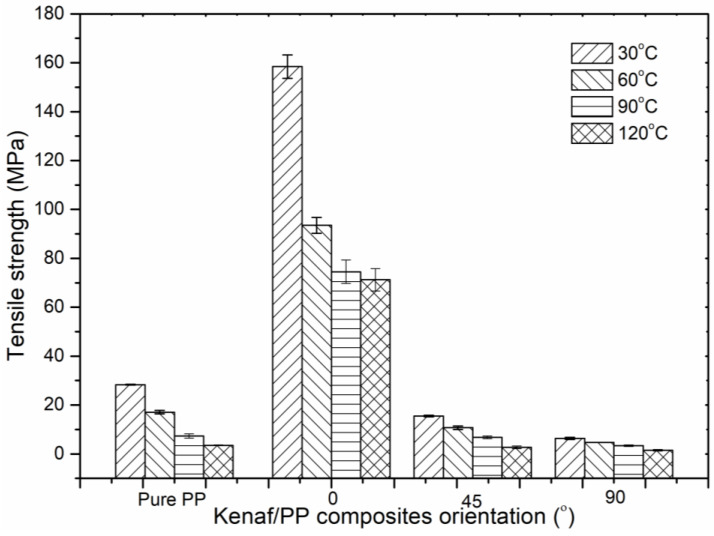
Tensile strength of kenaf/polypropylene (PP) composites at different orientation angles and temperatures.

**Figure 3:**
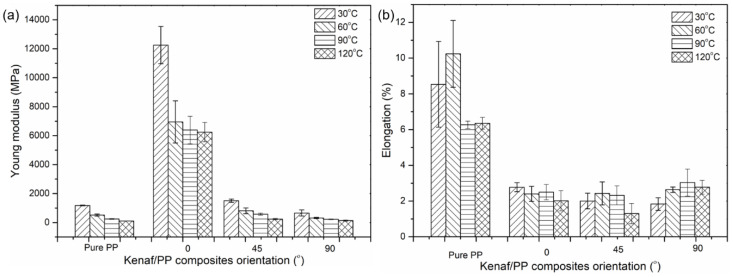
(**a**) Young’s modulus and (**b**) elongation of kenaf/polypropylene (PP) composites at different orientation angles and temperatures.

Figure 2 illustrates the tensile strength of pure PP and kenaf/PP composites at different orientation angles between 0° and 90° as temperature was increased from 30 to 120 °C. The study found that, as temperature increased, both the kenaf/PP composites and the polypropylene tended to deteriorate, which is explained by polypropylene’s nature, given that it began to experience an unstable chain as the temperature reached 100 °C [24]. By comparison, the kenaf/PP composites experienced an unstable structure when the temperature increased above 80 °C [25,26]. As shown in Figure 2, at the temperature range of 60 to 120 °C, the kenaf/PP composites were able to achieve a maximal tensile strength that ranged from 70 to 90 MPa, which is sufficient for automotive parts (e.g., a trunk cover or dashboard) that require a tensile strength of around 30 MPa [2,15]. These findings indicated that kenaf/PP composites are appropriate to be used, given that the applied temperature (60 to 120 °C) was near that of other commercial composite products used in high-temperature applications that can offer better mechanical properties [18]. Furthermore, Figure 2 demonstrates that, as the orientation angle increased from 0° to 45° and 90°, tensile strength dropped dramatically, from ~90 to ~20 MPa, even though theoretically, the fibers being aligned in one direction within the composite materials should enhance their mechanical properties [7,27]. This phenomenon occurred because, as the fibers were oriented to align with the applied load, the inner fiber layer was damaged, resulting in their mechanical properties being lowered, as shown in Figure 3a [28,29].

Theoretically, the aligned fibers that were perpendicular to the applied load required higher loading before the composite structure began to fail [30,31]. In contrast, the aligned fibers that were parallel showed a lower applied load resulting in fiber breakage [30,32]. This can easily be seen in Figure 1c,d, in which the failures obviously occurred and propagated along the kenaf fibers at orientation angles of 45° and 90°. This explains why the kenaf/PP composites that were aligned at 0° perpendicular to the applied load were able to sustain excellent tensile strength until 160 MPa compared to the kenaf fibers aligned at 45° and 90°. Moreover, previous studies suggested that, at an orientation angle of 0°, kenaf/PP composites possessed a much longer kenaf fiber structure due to minimal fiber breakage, thereby aiding in strengthening the composite structure by homogenously distributing the load [24,33,34]. Unlike with the fiber orientations of 45° and 90°, more kenaf fiber breakage was experienced, given the localized applied load that weakened the strength of the composite [35,36]. Lower tensile strength was also recorded for the pure PP when compared to the aligned kenaf/PP composites at 0° because of the existence of the aligned kenaf fibers, which strengthened the overall structure of the composite [35].

A similar trend can also be seen in Figure 3a, where the Young’s modulus of kenaf/PP composites appeared to decrease as temperature increased. In fact, some studies reported that the drop experienced by the kenaf/PP composites was mainly due to the warpage that occurred on the composite surface resulting from the crystallization of the polymer structure, causing brittleness [37,38]. Therefore, this could affect the mechanical properties of the kenaf/PP composites at temperatures above 60 °C [39]. Figure 3b shows that the elongation of kenaf/PP composites resulted in no significant change as temperature increased. Additionally, the orientation of the fibers did not affect the elongation of the kenaf/PP composites, with the average elongation recorded at 2.5%. However, this slightly differed from the elongation results obtained for the pure PP, where the most elongation was recorded at an average of 10%. These findings support the Young’s modulus and tensile-strength results, indicating that the kenaf/PP composites exhibited greater stiffness compared to that of pure PP [40].

**Figure 4:**
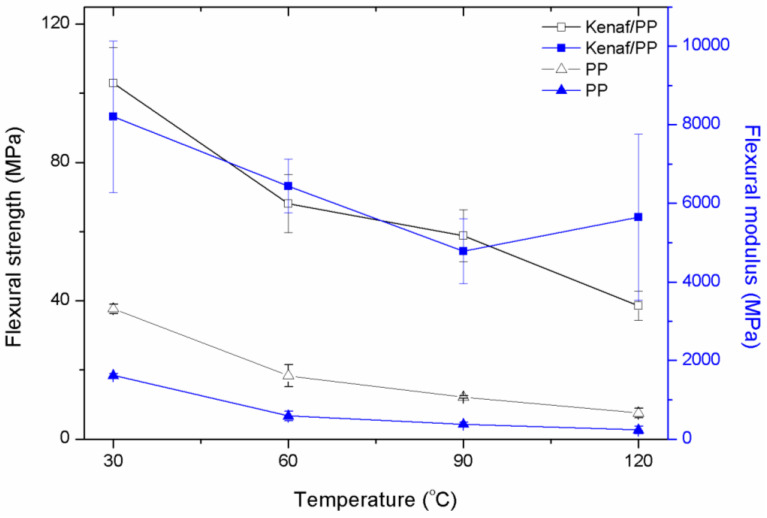
Flexural strength and flexural modulus of kenaf/PP composites at different temperatures.

Figure 4 illustrates the flexural strength of kenaf/PP composites, where the highest flexural strength was recorded as 105 MPa at 30 °C. However, as temperature increased to 120 °C, flexural strength began to decrease until reaching an average of 70 MPa. The same trend was recorded for pure PP, where flexural strength decreased as temperature increased. However, flexural strength recorded for the kenaf/PP composites was three times higher than that of the pure PP, at an average of 40 MPa. A similar trend was also observed in the flexural-modulus results, where the kenaf/PP composites exhibited excellent flexural strength and flexural modulus compared to pure PP. Past studies reported that the common flexural strength and flexural modulus recorded for a kenaf-composite-reinforced epoxy resin were, on average, 180 and 15 MPa, respectively [7,23]. Studies have also shown that the mechanical properties of fabricated aligned (0°) kenaf/PP composites at a temperature of 120 °C exceeded those of commercial products (Product T). Detailed results of the comparison between fabricated composites (0° orientation angle) and commercial products are listed in Table 2. Therefore, on this basis, the outstanding mechanical properties of kenaf/PP composites were comparable to those of commercial products, and have much to offer given their potential to withstand high-temperature operations while maintaining recyclability [41].

**Figure 5:**
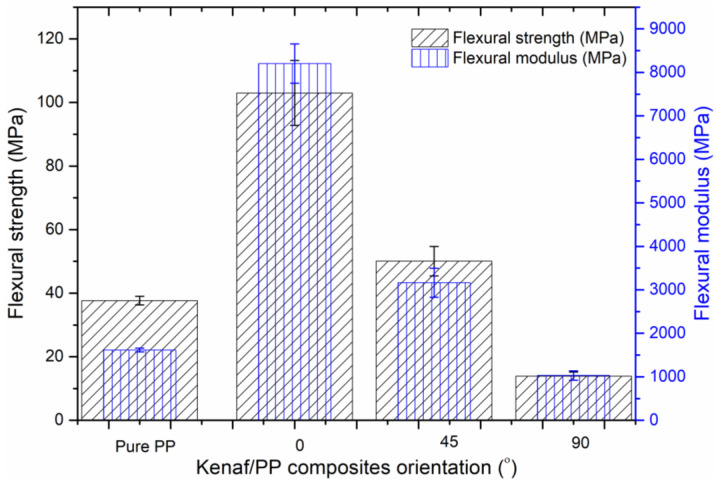
Flexural strength and flexural modulus of kenaf/polypropylene (PP) composites at different orientation angles at temperature of 120 °C.

The flexural strength and flexural modulus of the kenaf/PP composites at different orientation angles and room-temperature conditions are displayed in Figure 5. The aligned kenaf fibers at an orientation angle of 0° demonstrated maximal flexural strength and flexural modulus of 110 MPa and 8.5 GPa, respectively. Obtained results were six times greater than those of pure PP. Previous studies explained this result by reporting that, when kenaf fibers were aligned in a unidirectional position, they possessed the most stiffness because fiber efficiency was strong given that the fibers were able to contribute the same applied load [42]. On the other hand, when the kenaf fiber was oriented on 45° and 90° angles, both flexural strength and flexural modulus tended to deteriorate from 20 and 40 MPa to 1 and 3 GPa, respectively, which was marginally higher compared to the obtained tensile strength. Therefore, on this basis, the orientation of kenaf fibers influenced the flexural strength of the composite material by retaining the maximal load being applied [43,44].

**Figure 6:**
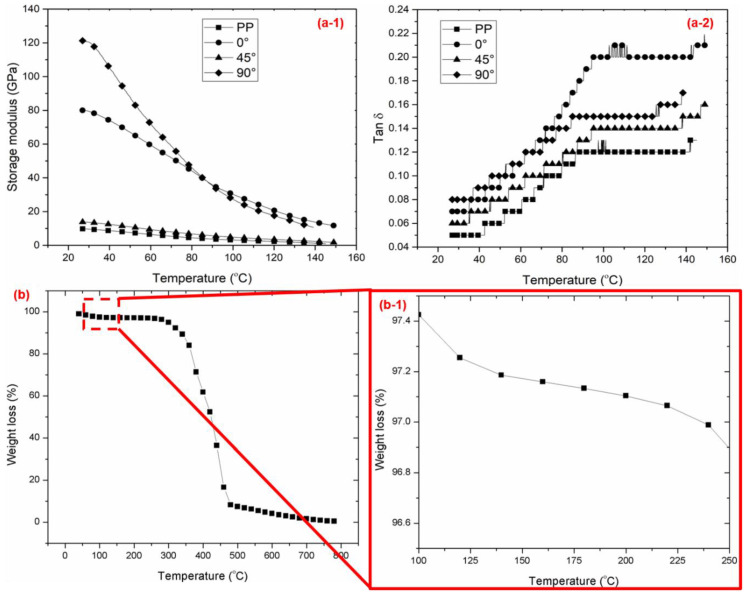
Dynamic mechanical analysis (DMA) of kenaf/PP composites. (**a1**) Storage modulus and (**a2**) tan δ at a temperature range of 30 and 150 °C, and (**b**) TGA of kenaf fibers; (**b1**) enlarged image of (**b**).

**Figure 7:**
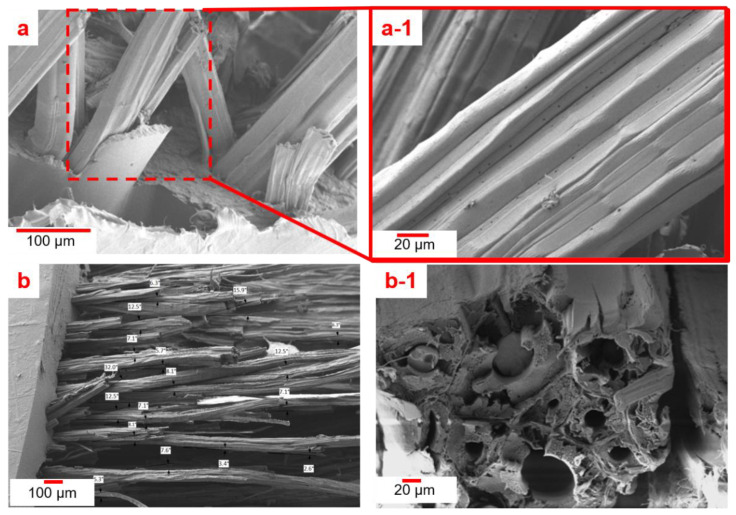
Images of (**a**) pulled-out kenaf fibers and (**a1**) smooth kenaf-fiber surface, (**b**) kenaf/PP composite at orientation angle of 90°, and (**b1**) kenaf/PP composite at orientation angle of 90° (cross-section).

The effects of fiber orientation and temperature changes were determined on the basis of DMA results, given that they provided information about the materials’ strength and brittleness related to the storage modulus, while tan δ (Figure 6a2) identified its elasticity behavior [45]. Figure 6a1 shows that the maximal storage modulus of 120 MPa was recorded at an orientation angle of 90°, followed by 80 MPa at 0°. This phenomenon was due to the kenaf composite at 90° behaving like an elastic material that stored more energy and less strength compared to the aligned kenaf composite at 0°. Meanwhile, for kenaf/PP composites at 45°, the storage modulus indicated the extreme energy loss contributed to the direction of the kenaf fibers, which were unable to withstand excessive amounts of energy. A similar negative trend was also shown in the kenaf/PP composites at 90°, which supported the lowest flexural strength recorded at a temperature of 120 °C. In addition, Figure 6a2 indicates that there were increments of tan δ due to the increase in interface bonding among the kenaf/PP composites, which later reduced the damping factor because of the decrease in mobility of the molecular chains at the kenaf/PP composites’ interface [45].These are shown in Figure 7b,b1, in which at 90°, the adhesion between the kenaf fibers and the matrix was low, with no reinforcement within the kenaf fibers as the applied load was only subjected to the polymer matrix. In contrast, compared to the 90° orientation of kenaf/PP composites, the composites at a 0° orientation angle seemed to experience no misalignment, improper wettability, or any other defect which might deteriorate their mechanical performance, which was also reported by another study using similar kenaf/PP composites [46]. This was because the prepared kenaf fibers were combed rather than chemically treated, further resulting in a balanced mixture and excellent composite structure [46]. Furthermore, Figure 7a,a1 demonstrates that the kenaf fibers experienced a fiber pull-out due to strong adhesion within the kenaf fibers and matrix. However, increasing the temperature resulted in the kenaf composite behaving like a viscous material, leading to energy dissipation. This further resulted in higher stress being required to break the material bonding, which lowered its storage modulus. Prior research exhibited that kenaf fibers tend to experience 9.5% moisture evaporation at a temperature of 153 °C, which was supported by these findings: as temperature increased to 120 °C, the bonding of the kenaf composite was weakened, as higher stress was required to break it [47]. Figure 6b,b1 shows that, as the temperature exceeded 120 °C, kenaf fibers started to deteriorate and degrade, resulting in the composite bonding weakening. This explains the results that were obtained with an orientation angle of 0°, where at 100 °C (the melting temperature at which there was complete strength loss), the storage modulus was recorded at 40 MPa compared to 35 MPa for an orientation angle of 90°. The curve also illustrates that pure PP and kenaf/PP at an orientation angle of 45° experienced no significant change, indicating that there is marginal difference in both of the materials’ thermomechanical properties [23]. Compared to the pure kenaf fiber shown in Figure 6b, thermogravimetric analysis of kenaf/PP composites reported that, in the temperature range of 30 to 175 °C, endothermic peaks appeared at ~85 °C [41]. Studies using similar kenaf/PP composites reported that exothermic peaks were recorded at the temperature of 250 °C due to the degradation and decomposition of cellulose at higher temperatures [41]. Furthermore, Figure 6 displays that the tan δ value increased as temperature increased. The maximal tan δ value recorded at the 0° orientation angle, 0.21, indicated that the aligned kenaf fibers at 0° exhibited a rigid composite structure (being nonthermoplastic). Indeed, this contrasted with the results obtained for pure PP, which were the lowest recorded at 0.05; this indicates that it is a purely elastic material. Therefore, on this basis, the dynamic mechanical behavior of kenaf/PP composites was dominated by the polypropylene resin rather than by kenaf fibers [23].

**Figure 8:**
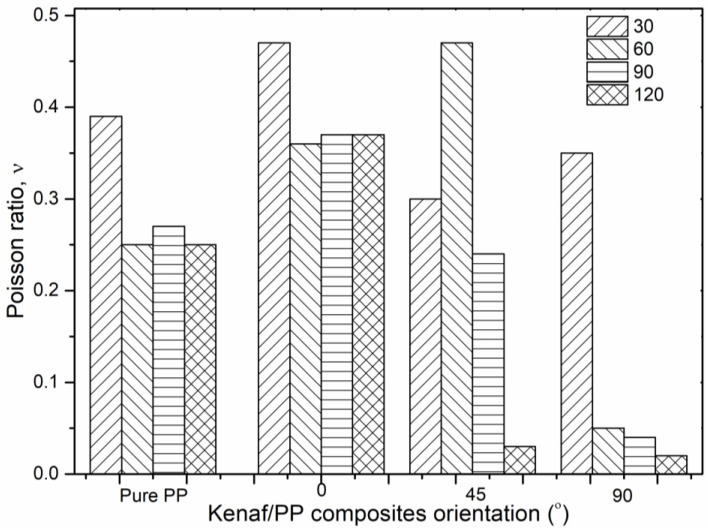
Poisson’s ratio results of kenaf/polypropylene (PP) composites at various temperatures and orientation angles.

Figure 8 displays Poisson’s ratio for the kenaf/PP composites, where the maximal values were obtained at orientation angles of 0° and 90°, with 0.47 and 0.35, respectively, at a temperature of 30 °C. At an orientation angle of 45°, the maximal Poisson’s ratio was recorded at 0.47 at a temperature of 60 °C. Therefore, on the basis of the histogram results, the kenaf/PP composites at an orientation angle of 0° attained the most consistent result, with no dramatic change, even though the temperature rose to 120 °C. This was in contrast with kenaf/PP composites at orientation angles of 45° and 90°, which experienced a dramatic drop as temperature increased to 120 °C. These findings were supported by previous studies of epoxy resin, which reported that Poisson’s ratio increased at a marginally lower temperature [48]. Therefore, on this basis, the composite at an orientation angle of 0° offered similar elasticity behavior to that of pure PP. This indicated that kenaf/PP composites can attain high mechanical strength, as proven in the tensile-strength results (Figure 2). Furthermore, these findings also yielded excellent Poisson’s ratio values, which were above the average obtained value (0.2–0.3) by other studies involving kenaf/polypropylene materials.

## 4. Conclusions

This study investigated aligned kenaf/PP composites at orientation angles between 0°, 45°, and 90°, and prepared through a combing process at temperatures ranging from 30 to 120 °C. The findings indicated that the aligned kenaf fibers at an orientation angle of 0° demonstrated excellent mechanical properties, with a tensile strength of ~90 MPa at a temperature of 120 °C. These findings were supported by the flexural-strength results, in which fibers at an orientation angle of 0° obtained ~50 MPa, which was above the average value (~30 MPa) of a commercial automotive product at a temperature of 120 °C. Accordingly, the study found that the mechanical properties of fabricated kenaf/PP composites aligned at an orientation angle of 0° were able to exceed those of commercial products in today’s market. Therefore, these results demonstrated that kenaf/PP composites have so much to offer in terms of affordability, excellent mechanical properties relating to safety issues, and lightweight components, especially in automotive applications. The development structure and kenaf-fiber content should be addressed, along with the calculation of the volume-void content, in future investigations.

## Figures and Tables

**Table 1:** Composition of kenaf fibers.

Composition	Percentage (%)
Extractive	5.88
Holocellulose (cellulose +hemicellulose)	96.17
α-Cellulose	61.02
Hemicellulose	35.15
Lignin	12.5

**Table 2:** Comparison of mechanical properties of fabricated kenaf/PP composites and Product T at 120 °C.

Mechanical Properties	Kenaf/PP (0°) at 120 °C	Product T
Tensile strength (MPa)	~90	30.52
Flexural strength (MPa)	~50	39.4
Young’s modulus (GPa)	~7.5	2.56
Flexural modulus (GPa)	~6.0	4.51
